# Supplementary material for: Dynamics of bacterial population growth in biofilms resemble spatial and structural aspects of urbanization
Source: Nat Commun. 2020 Mar 13;11:1354. doi: 10.1038/s41467-020-15165-4 (PMC7070081; doi:10.1038/s41467-020-15165-4)
Supplement: Supplementary file 1 — Supplementary Information [file 41467_2020_15165_MOESM1_ESM.pdf]

SUPPLEMENTARY INFORMATION

for

Dynamics of bacterial population growth in biofilms resemble spatial and structural aspects of urbanization

Amauri J. Paula[1,2,*] Geelsu Hwang,[2,3,4,*] Hyun Koo[2,4*]

[1]Solid-Biological Interface Group (SolBIN), Department of Physics, Universidade Federal doCeará, P.O. Box 6030, 60455-900, Fortaleza-CE, Brazil

[2]Biofilm Research Labs, Levy Center for Oral Health, Department of Orthodontics, Divisions of Pediatric Dentistry and Community Oral Health, School of Dental Medicine, University of Pennsylvania, Philadelphia-PA, 19104, USA

[3]Department of Preventive and Restorative Sciences, School of Dental Medicine, University of Pennsylvania, PA, USA

[4]Center for Innovation & Precision Dentistry, School of Dental Medicine, University of Pennsylvania, Philadelphia, PA 19104, USA

Corresponding Author

* Phone.: +55 85 3366 9270; email: amaurijp@gmail.com

* Phone.: +1 215 573 0994; email: geelsuh@upenn.edu

* Phone.: + 1 215 898 8993; email: koohy@upenn.edu

Keywords: *biofilm, growth law, dynamics, EPS, Streptococcus mutans, Candida albicans, fluorescence imaging, image processing, 4D analysis, surface topography, competition, symbiosis.*

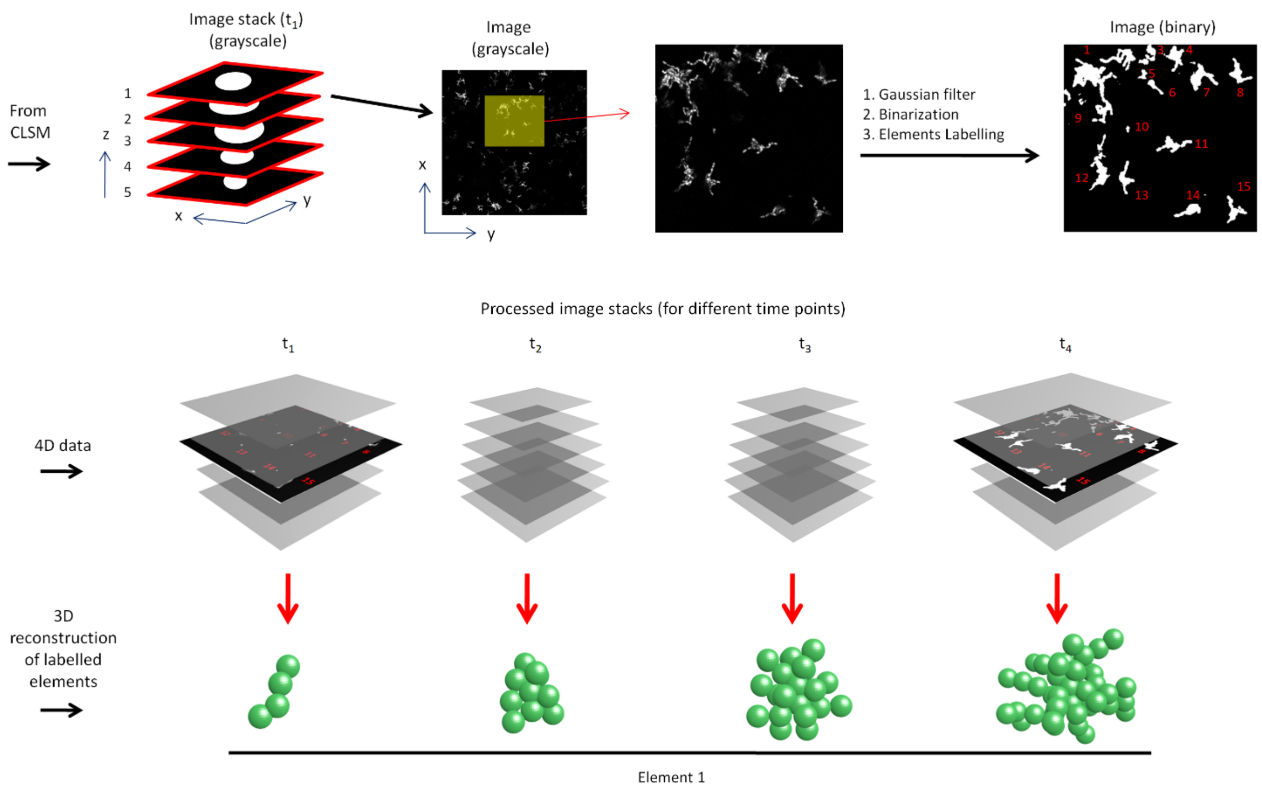

Supplementary Figure 1. Diagram depicting the image processing steps performed for CLSM image stacks. Time-resolved image stacks (4D data) were processed with the sequential use of 3D Gaussian filter, image binarization process and elements labeling. Elements (i.e. microbes) were further reconstructed from the image stacks. Finally, elements convex hull and volume values were determined and growth curves were plotted (i.e., volumes vs. time).

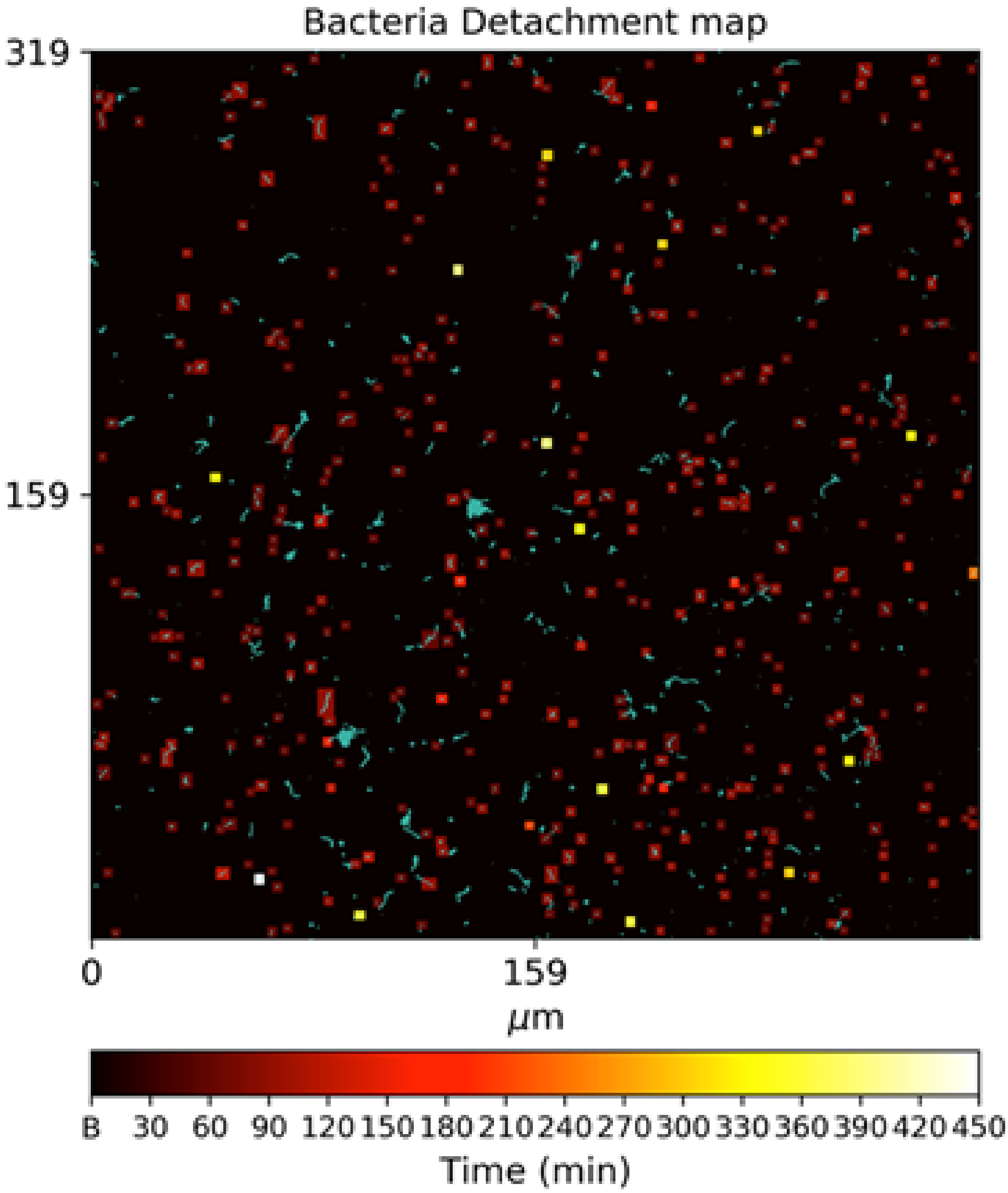

Supplementary Figure 2. Time-lapsed bacteria detachment map. Map indicating the time bacteria were removed from the surface after starting the flow (t > 0). Shapes in light blue represent bacteria signal at t0 (projection from the 3D image stack). 'B' in the time scale stands for background. Approximately 40% of colonizing cells detached from HAD surface.

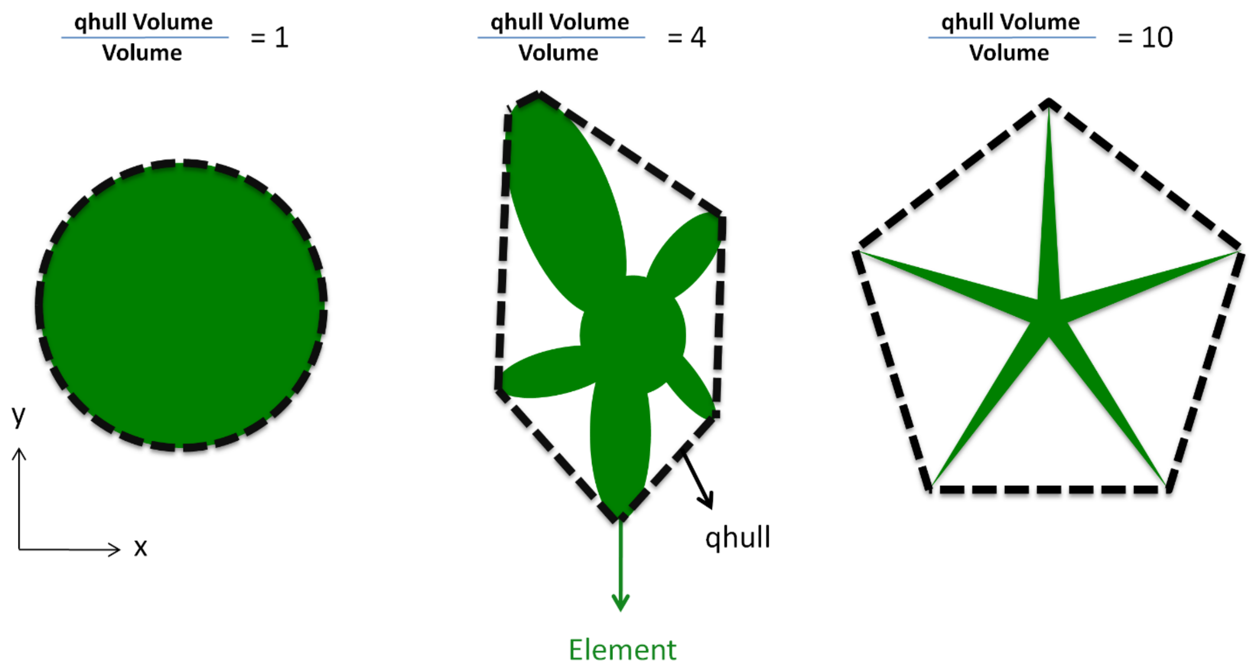

Supplementary Figure 3. Convex hull representation. Diagram representing the variation of the (qhull Volume / Volume) ratio as a function of the shape/morphology of the elements found in the CLSM image stacks (i.e., chains, clusters, aggregates, and microcolonies). The larger the ratio the larger is the deviation from a spherical morphology, a result of a more branched morphology.

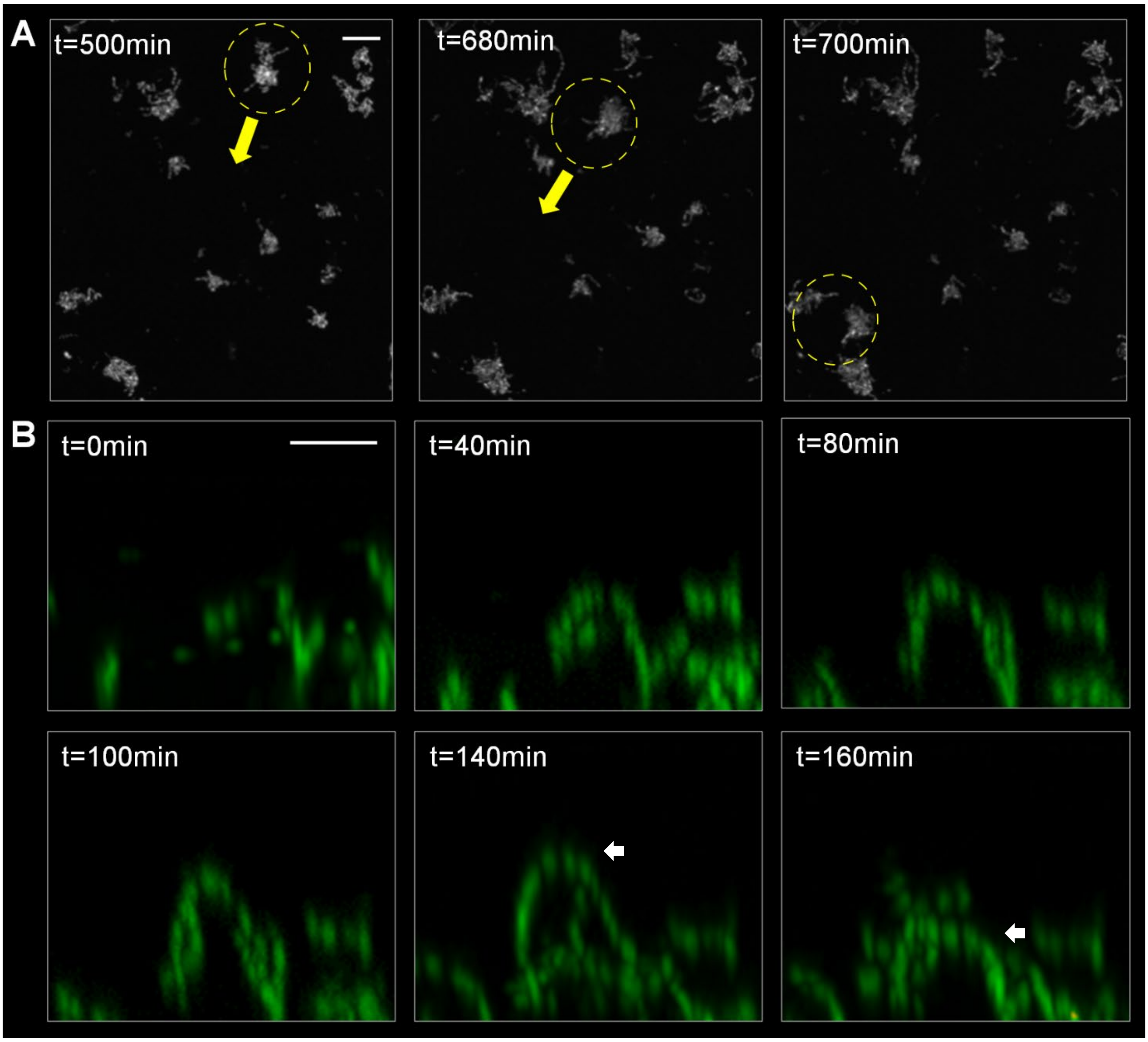

Supplementary Figure 4. Confocal laser-scanning microscopy (CLSM) image projections showing compromised strucural stability of *S. mutans* biofilms or cell chains when EPS matrix is degraded or absent. (A) Z-projection (max intensity) of the CLSM images stack of *S. mutans* growing on HAD in 1 %-w/v sucrose with the presence of EPS-degrading dextranase and mutanase (1:1 0.18U mL^{-1}) under flow. Yellow circle and arrows indicate detachment of the highlighted bacterial cluster from the surface and further movement under flow, indicating disruption of adherence (unstable communities). **(B)** Time-lapsed cross-section image sequence of *S. mutans* at a single-cell level growing in 0.5%-w/v glucose + 0.5%-w/v fructose (which can not be used as substrate for EPS glucan synthesis). The sequence demonstrates the structural instability of the bacterial cell-chain.

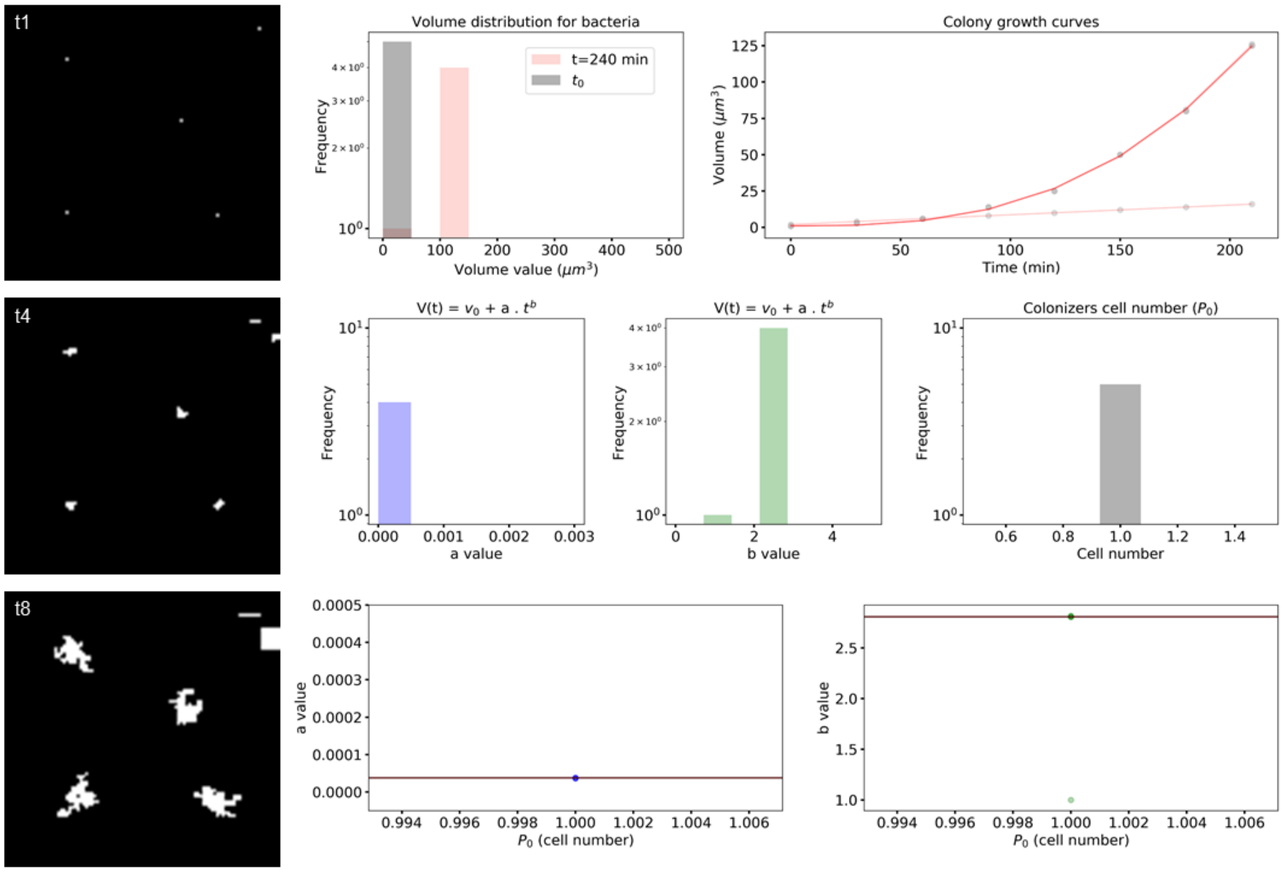

Supplementary Figure 5. Validation of BioSPA algorithm. Collection and fitting performance using a mock dataset containing biofilm objects. In the TestSet, a population of 6 colonizers growing by different growth laws were generated. First, five colonizers were generated with a growth law where b exponent is 2.80 and the standard deviation (STD) of the fitting is 0.06 to mimic Dynamic Colonizers. Among these five, one colonizer was generated at the edge of the image stack to confirm the algorithm cropping routine: colonizers located at the edge are excluded from the population analysis. The standard deviation of 0.06 was used to confirm the algorithm fitting-cropping routine: colonizers not following power-law (STD > 0.1) are excluded from the population analysis and they are considered as "non-fitted" (see Figure 4b). Finally, one colonizer was generated with a growth law where b exponent is 1.0 (STD of the fitting is 0) to confirm perfect fitting. All colonizers started with an initial volume of 1 μm^3 (representing 1 cell).
